# Loss and Gain of Function in *SERPINB11*: An Example of a Gene under Selection on Standing Variation, with Implications for Host-Pathogen Interactions

**DOI:** 10.1371/journal.pone.0032518

**Published:** 2012-02-29

**Authors:** Susana Seixas, Nevyana Ivanova, Zelia Ferreira, Jorge Rocha, Bruno L. Victor

**Affiliations:** 1 Institute of Molecular Pathology and Immunology of the University of Porto (IPATIMUP), Porto, Portugal; 2 Department of Zoology and Anthropology, Faculty of Sciences, University of Porto, Porto, Portugal; 3 Instituto de Tecnologia Química e Biológica, Universidade Nova de Lisboa, Oeiras, Portugal; Oxford University, United Kingdom

## Abstract

Serine protease inhibitors (SERPINs) are crucial in the regulation of diverse biological processes including inflammation and immune response. *SERPINB11*, located in the 18q21 gene cluster, is a polymorphic gene/pseudogene coding for a non-inhibitory SERPIN. In a genome-wide scan for recent selection, *SERPINB11* was identified as a potential candidate gene for adaptive evolution in Yoruba. The present study sought a better understanding of the evolutionary history of *SERPINB11*, with special focus on evaluating its selective signature. Through the resequencing of coding and noncoding regions of *SERPINB11* in 20 Yorubans and analyzing primate orthologous sequences, we identified a full-length *SERPINB11* variant encoding a non-inhibitory SERPIN as the putative candidate of selection – probably driven to higher frequencies by an adaptive response using preexisting variation. In addition, we detected contrasting evolutionary features of *SERPINB11* in primates: While primate phylogeny as a whole is under purifying selection, the human lineage shows evidence of positive selection in a few codons, all associated with the active *SERPINB11*. Comparative modeling studies suggest that positively selected codons reduce SERPINB11's ability to undergo the conformational changes typical of inhibitory SERPINs – suggesting that it is evolving towards a new non-inhibitory function in humans. Significant correlations between *SERPINB11* variants and the environmental variables, pastoralism and pathogen richness, have led us to propose a selective advantage through host-pathogen interactions, possibly linked to an adaptive response combating the emergence of infectious diseases in recent human evolution. This work represents the first description of a resurrected gene in humans, and may well exemplify selection on standing variation triggered by drastic ecological shifts.

## Introduction

Serine protease inhibitors (SERPINs), a superfamily of proteins found in all domains of life (*Eukarya*, *Eubacteria*, and *Archaea*), have preserved their tertiary structure throughout evolution. Typically, SERPINs neutralize serine or cysteine proteases by a unique suicide substrate-like inhibitory mechanism that entails a dramatic rearrangement in protein folding. SERPINs are able to entrap proteases by presenting a pseudosubstrate in an exposed reactive center loop (RCL). Upon RCL cleavage, SERPINs initiate a major conformational change from “stressed” to “relaxed” (S-to-R – transition) leading to distortion and permanent inactivation of the protease catalytic site [1]–[4].

In vertebrates, the vast majority of SERPINs are important in regulating proteolytic cascades in biological processes such as blood coagulation, development, apoptosis, and inflammation. However, a small fraction of these proteins have lost their inhibitory activity and developed other functions as hormone carriers, chaperones, or storage proteins [3], [4]. The 37 SERPINs known in humans belong to nine phylogenetic clades (A–I) defined by similarities in protein sequence and gene structure [2], [5]–[7].

**Figure 1:**
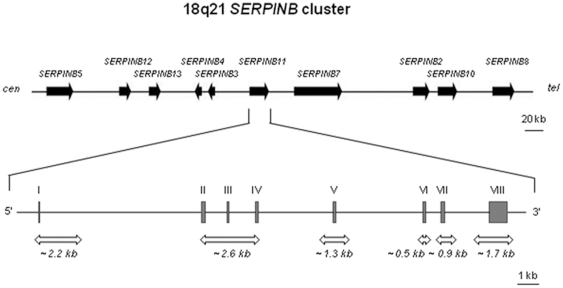
Schematic representation of the18q21 *SERPINB* gene cluster. Upper diagram shows the relative position of the *SERPINB* genes in the cluster and lower diagram shows *SERPINB11* gene organization (exons are represented by grey boxes). Large white arrows indicate the extent of segments surveyed in the resequencing study of the YRI population.

Clade B SERPINs, also called ov-serpins due to their high sequence similarity to chicken ovalbumin, are located in two clusters: *SERPINB1*, *B6*, and *B9* are located in the chromosome 6p25 region, and *SERPINB2*,*B3*, *B4*, *B5*, *B7*, *B8*, *B10*, *B11*, *B12*, and *B13* are located in the 18q21 region (Figure 1) [8], [9]. SERPINBs differ in several respects from all other SERPINs. While most SERPINs exert their function as extracellular proteins, SERPINBs are found predominantly within cytoplasmic or nuclear cell compartments, where they are thought to protect against promiscuous proteolysis [10]–[12]. Indeed, several clade B members are known to respond to inflammatory mediators, to be involved in leukocyte development, and to participate in phagocytosis through degradation of bacterial components [11], [13], [14].

*SERPINB11* is located in the 18q21 cluster and, based on its low sequence identity (less than 50%) with other clade B SERPINs, it is likely to represent an ancestral duplicate. An investigation of the activity of *SERPINB11* identified two major gene transcripts: One corresponds to a full-length product and codes for a regular SERPIN; the other carries a premature stop codon at position 90, which results in a nonfunctional variant (pseudogene) [15]. Furthermore, a series of biochemical assays demonstrated that SERPINB11 had lost its ability to inhibit trypsin-like proteases – possibly due to accumulation of nonconserved amino acid replacements outside the RCL region [15]. Interestingly, in a human genome-wide scan (GWS) for recent positive selection using HapMap phase II data and the integrated haplotype score (iHS;, a linkage disequilibrium (LD) - based statistic [16]), *SERPINB11* was identified as a potential candidate gene. *SERPINB11* yielded a significant p-value (0.041) in the Yoruba, from Ibadan, Nigeria (YRI), indicating that SERPINB11 has a high proportion of significant single nucleotide polymorphisms (SNPs) (|iHS|>2) compared with other genes, and placing *SERPINB11* above the top 5% of the empirical genome-wide distribution from the YRI population [16].

The current study sought a deeper understanding of the evolutionary history of *SERPINB11*, with a special focus on the signature of selection identified in the YRI. Our approaches included analyzing HapMap phase II haplotype data, resequencing *SERPINB11* in 20 YRI individuals, and surveying seven nonhuman primate sequences. Statistical tests enable us to: identify a long-range haplotype carrying six functional variants; confirm a non-neutral evolution of *SERPINB11*; and contrast the overall levels of constraints in *SERPINB11* with the evidence of selection in humans, favoring a few codons predicted to affect both protein structure and stability. Collectively, our results point to a full-length *SERPINB11* variant encoding a non-inhibitory SERPIN as the putative target of selection, probably resulting from an adaptive response based on preexisting variation.

## Results

### Evaluation of the selective signature based on HapMap phase II data

**Figure 2:**
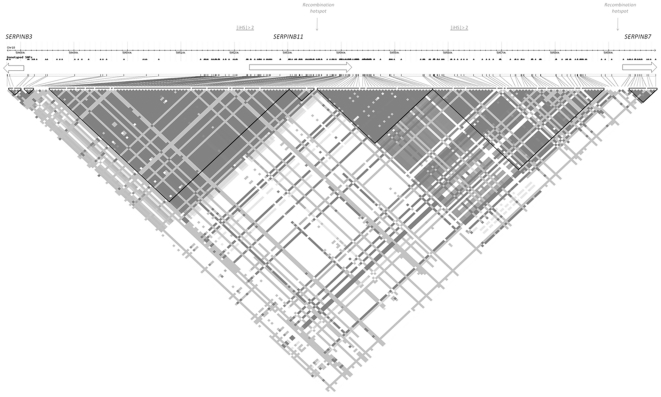
LD plot of HapMap phase II YRI data centered on the *SERPINB11* region. The image was constructed using *Haploview* 4.1 software. The triangular units designate LD blocks. The degree of LD between pairs of markers is indicated by the |D′| statistic (|D′| = 1, black; |D′|>1, shades of grey) (Higher resolution figure is provided as supplementary material – Figure S1).

In the database from a GWS for recent positive selection based on the iHS statistic [16] and relying on HapMap phase II data, we found a significant p-value (p = 0.040607) for *SERPINB11* in the YRI. Low empirical gene p-values are frequently associated with clumps of SNPs with significant iHS scores (|iHS|>2) and long haplotypes [16], [17]. In this case, 34 SNPs with significant iHS scores (File S1) were identified the vicinity (200 kb window) of *SERPINB11*. These SNPs were organized into two major clusters and located in distinct LD blocks; the first cluster, occupied a 34-kb block encompassing a large *SERPINB11* segment; the second cluster was in a 30-kb block downstream of *SERPINB11* (Figure 2, Figure S1 and File S1). According to local recombination inferences [18], [19], a hotspot is included within *SERPINB11* (39 cM/Mb), spliting the region into areas of strong LD that contain the two clusters of SNPs with significant iHS scores (Figure 2, Figure S1 and File S1).

To define long haplotypes carrying the potential selected variants, we used SNP iHS values to identify configurations of tightly linked alleles [16], [17], [20]. This approach led to the recognition of two neighboring haplotypes, one with a ∼60% frequency and bearing the E90 allele (active gene) and another with a ∼80% frequency and no clear association with a known functional variant. Approximately 40% of the chromosomes could be united in a single long-range haplotype (>80 kb) that spans the recombination hotspot and encompasses the full *SERPINB11* sequence (File S1).

### *SERPINB11* sequence variation and structure

**Figure 3:**
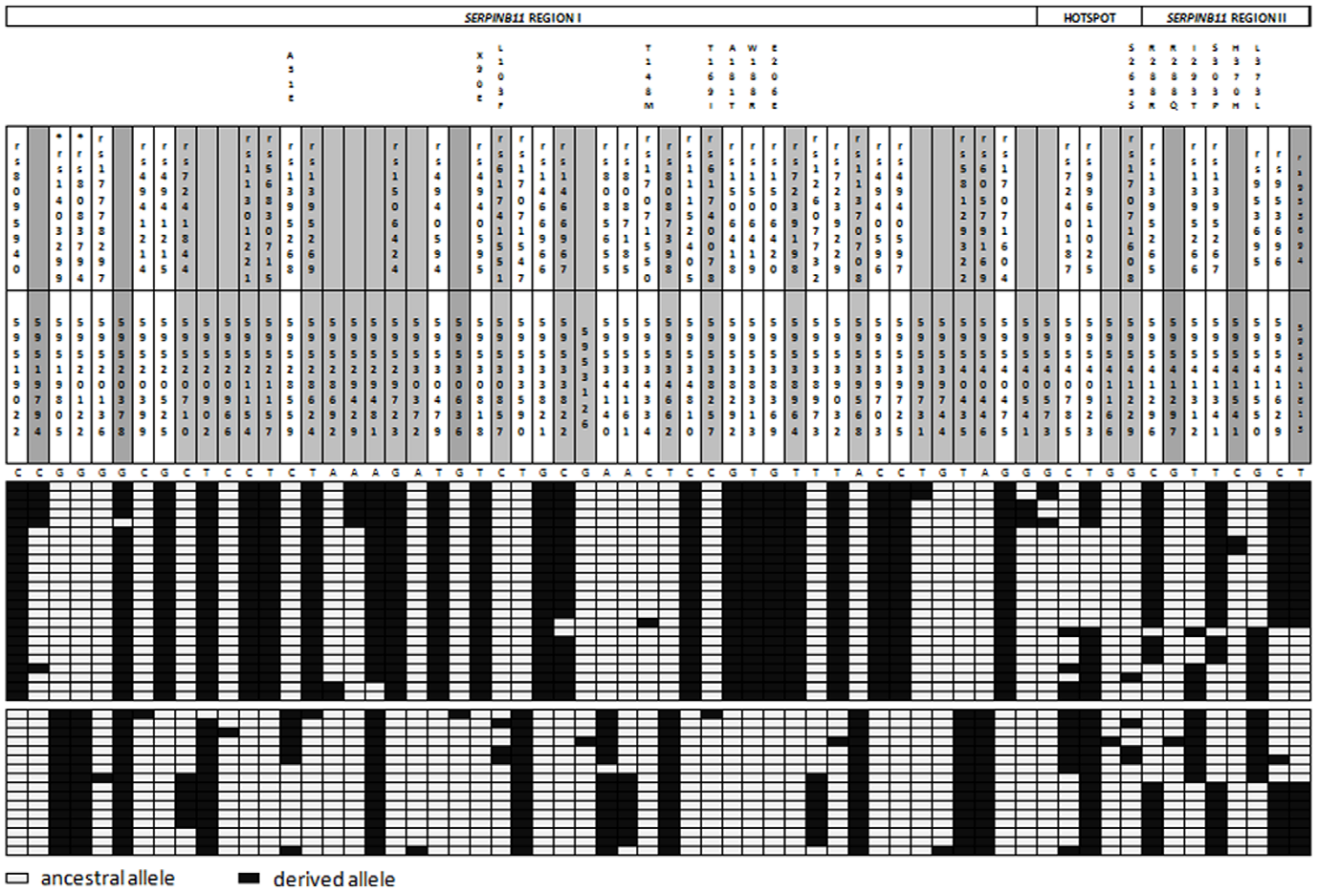
Haplotypes as inferred by PHASE2 for *SERPINB11*. Orthologue nonhuman primate sequences were used to infer the ancestral state at each site. SNPs typed in HapMap phaseII are shown on a white background; SNPs not typed by HapMap are shown on a grey background. Numbers indicate the chromosome position of each polymorphic site, based on a NC000018 reference sequence. Non-synonymous and synonymous sites are labeled. SNPs with a significant iHS statistic are marked by an asterisk.

To reveal the complete functional variation of *SERPINB11*, we surveyed six fragments encompassing a total of 9.2 kb (Figure 1), from a subset of 20 YRI individuals. A total of 62 polymorphic sites were identified (Figure 3), including the nonsense mutation X90E, 9 non-synonymous replacements (A51E, L103F, T148M, T169I, A181T, W188R, R288Q, I 293T, and S303P), 5 synonymous substitutions, and 47 noncoding polymorphisms. Except for the L103F, T169I, and R288Q variants, all non-synonymous mutations were previously described by Askew and colleagues [15]. Furthermore, in the 3′ untranslated region, rs953696T and rs953694C alleles were predicted to generate binding sites for microRNAs (miRs): rs953696T for *hsa-mir-1302-8* and *has-mir-1200*, and rs953694C for *hsa-mir-1302-2* (miRBase – http://www.mirbase.org/).

In the upstream region of the recombination hotspot (*SERPINB11* Region I; Figure 3) the substitutions X90E, A181T, and W188R were found in complete LD (|D′| = 1 and r^2^ = 1) with rs1403299 and rs8083794 sites belonging to the cluster of SNPs with significant iHS scores, and. In the region downstream of the hotspot (*SERPINB11* Region II; Figure 3) strong levels of LD were also detected for rs953696 and rs953694 sites and for the S303P replacement (|D′| = 1; r^2^≥0.90). Interestingly, the six functional alleles E90, T181, R188, P303, rs953696T, and rs953694C were associated with a long-range haplotype at a frequency of approximately 40% extending over the recombination hotspot (Figure 3).

### Polymorphism levels and neutrality tests

Statistics from the polymorphism data for *SERPINB11* are shown in Table 1. Tajima's D statistic summarizes the information about the spectrum of allele frequencies [21] and in populations of African descent, tends to be slightly negative because of a small excess of rare variants [22]–[26]. However, the estimate obtained for *SERPINB11* in the YRI (Tajima's D = 1.42) differs from the common trend in populations of African descent and suggests an excess of intermediate frequency variants for both regions flanking the recombination hotspot (Table 1). The theoretical null distributions generated by coalescent simulations for a calibrated model of YRI demography [27] confirm that *SERPINB11* departs significantly from expectations under the neutral equilibrium model This condition is further sustained by alternative models of human demography (Table 2).

**Table 1:** Summary Statistics of Population Variation.

Population	N^a^	L^b^	S^c^	π^d^	θ_W_ e	D^f^	ρ^g^
***YRI: Yoruba from Ibadan in Nigeria***							
*SERPINB11*	40	9209	62	22.06	15.83	1.42	0.94
*SERPINB11 Region I*	40	7675	49	21.02	15.01	1.43	0.21
*SERPINB11 Region II*	40	827	8	35.83	22.74	1.64	8.61

a N – number of chromosomes.

b L – total number of sites surveyed.

c S – number of segregating sites.

d π – Nucleotide diversity per base pair (×10^4^).

e θ_W_ – Population mutation rate parameter: Watterson's estimator of θ (4Neμ) [94] per base pair (×10^4^).

f D – Tajima's D statistic [21].

g ρ – Population recombination rate parameter: Hudson's estimator of ρ (4N_e_r) per base pair (×10^4^), based on a conversion-to-crossover ratio of 2 and a mean conversion tract length of 500 bp [23], [95].

**Table 2:** The 97.5^th^ percentile of the null distributions generated by coalescent simulations.

Demographic model	Value of π	Value of Tajima's D
	(97.5^th^ percentile)	(97.5^th^ percentile)
**Constant size**	22.11*	1.61
**Recent Expansion** a		
(*N_0_* = 10^4^; *N_1_* = 10^7^; and *t* = 1000 g)	15.64*	1.27*
**Two-fold Growth** b		
(*N_0_*∼10^4^; and *t* = 1000 g)	16.39*	1.53
**Short and Severe Bottleneck** b		
(*N_0_*∼10^4^; *t_0_* = 1600 g; *b* = 0.1; *t_1_* = 1200 g)	23.22	2.14
**Long and Mild Bottleneck** b		
(*N_0_*∼10^4^; *t_0_* = 1600 g; *b* = 0.4; *t_1_* = 1200 g)	18.49*	0.96*
**Structure** a		
(*npop = 2* and *m = 1.0*)	22.06*	1.85
**Structure** c		
(*npop = 2* and *m = 0.5*)	23.63	2.23
**Best fit** d	18.70*	1.01*

a Model from Wang and colleagues [64];

b Model from Voight and colleagues [26];

c Model from Sabeti and colleagues [75];

d Model from Schaffner and colleagues [27];

*N* – effective size; *t* – time in generations; *b* – bottleneck intensity; *npop* –number of populations; *m* – migration rate per generation.

*Statistically significant – the observed statistic is higher than the 97.5^th^ percentile values.

The data set from the SeattleSNPs project (http://pga.gs.washington.edu/) captures the genetic variation of 316 genes with an established or predicted link to the human inflammatory response and provides an empirical comparison with the fit of *SERPINB11* to the global patterns of African variation. The detection of very few other genes with statistics higher than those for *SERPINB11* corroborates the previous finding of the outstanding nature of *SERPINB11* (Figure S2).

### Gene Genealogy and Age Estimates

**Figure 4:**
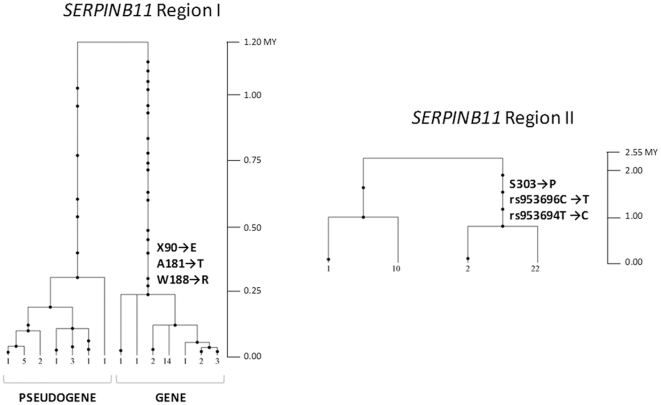
*SERPINB11* genealogies as estimated by GENETREE. Time is scaled in millions of years (MY). The indicated tree branches correspond to functional variants. Solid circles represent nucleotide substitutions. The numbers below the trees represent the numbers of each haplotype. In Region I, a Ne = 6,400 was calculated and 3 incompatible sites and 2 haplotypes were removed from the analysis; in Region II, a Ne = 14,800 was calculated and 5 incompatible haplotypes were removed.

To define the time frame of *SERPINB11* haplotypes, we used a coalescent analysis [28] to reconstructed the genealogies of regions I and II flanking the recombination hotspot. The resulting trees are represented in Figure 4; in both cases, we detected atypical tree structures, dominated by two deep-rooted branches. Such topologies are frequently regarded as evidence of long-term balancing selection or ancestral substructure, generally associated with time to most recent common ancestor (T_MRCA_) estimates ranging from 2 to 3 million years (MY) [29]–[33]. However, for region I, the 1.21±0.17 MY estimate fully agrees with both observed and expected T_MRCA_ from human autosomal genes [34]. In addition, the age estimate of the E90 allele (0.24±0.07 MY) suggests a relatively recent arising of the *SERPINB11* gene, near the time of origin of moderns humans and long after the appearance of P303, rs953696, and rs953694 alleles (0.88±0.44 MY). Nonetheless, when the T_MRCA_ of the full-length *SERPINB11* variant was calculated using the decay of haplotype sharing (DHS), a statistical method that exploits the breaking of haplotypes by recombination in succeeding generations, the estimated time of origin was 16,500 years ago, with a minimum estimate of 8,500 years.

### Population patterns and correlation with environmental variables

**Figure 5:**
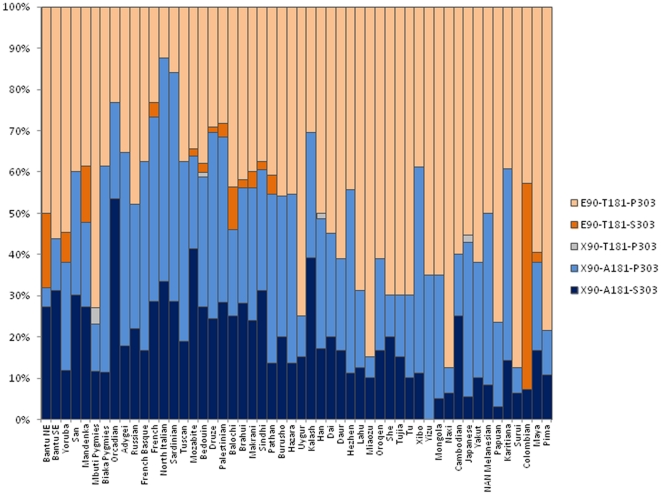
Worldwide distribution of common *SERPINB11* haplotypes as inferred by PHASE2 for the HGDP data [**35**].

To gain greater insight into *SERPINB11* variation, we cross-compared the patterns from three populations included in the HapMap Phase II project: Africans (YRI), Europeans (CEU: Utah residents with northern and western European ancestry), and Asians (CBH+JPT: Han Chinese from Beijing, China and Japanese from Tokyo, Japan) (Figure S3 and File S2). Importantly, we could identify sites X90E, A181T, and S303P as surrogate markers of the full-length *SERPINB11* haplotype in the HapMap data set (YRI: 40%; CEU: 28%; and CHB+JPT: 37%). The same sites were used to access the geographic distribution of *SERPINB11* haplotypes in the 52 worldwide populations from the Human Genome Diversity Project (HGDP) panel in Figure 5 [35].

**Figure 6:**
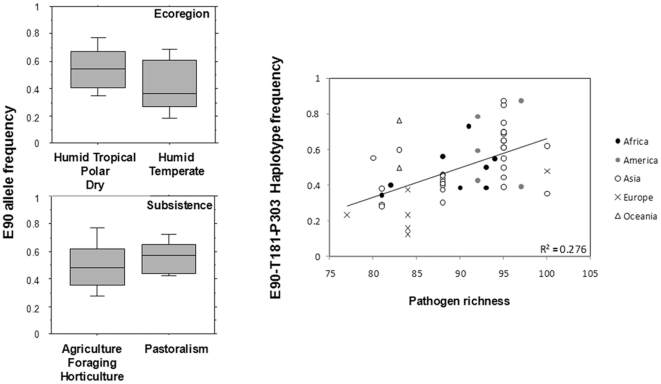
Association of *SERPINB11* worldwide variability with environmental variables. The data for categories of variables: Ecoregion and Subsistence data were entirely obtained from the database dbCline (http://genapps.uchicago.edu/labweb/index.html). The data for pathogen richness were collected from the GIDEON database (http://gideononline.com) and Li and colleagues [35] Populations are designated by their continent of origin. R^2^ indicates the correlation parameter.

We also inquired whether the observed worldwide functional *SERPINB11* variability [A51E (rs1395268), X90E (rs4940595), A181T (rs1506418), and S303P (rs1395267)] [35] could be connected with any environmentally dependent variable. To assess the impact of the variables: ecoregion (dry, polar, humid-temperate, and humid-tropical), subsistence (agriculture, foraging, horticulture, and pastoralism), and main dietary component (cereals, fats-meat-and-milk, and roots-and-tubers) on *SERPINB11* frequencies, we used a novel statistic, a Bayes factor, implemented through the dbCline database (http://genapps.uchicago.edu/labweb/index.html). This novel statistic measures the support for a model in which the allele frequencies of a SNP are dependent on an environmental variable in addition to population structure, compared to a model in which allele frequencies are dependent solely on population structure [36], [37]. Interestingly, the distributions of allele frequencies were significantly associated with pastoralism (X90E and A181T; p<0.009; Figure 6) and humid-temperate (X90E; p<0.033; Figure 6) variables. Next, to assess the impact of host-pathogen interactions on *SERPINB11* variability, we calculated the pathogen-richness parameter [33], [38] for intracellular pathogens. With the exception of A51E, all *SERPINB11* polymorphisms (X90E, A181T, and S303P) presented ranks equivalent to the 26 SNPs previously shown to have the strongest associations with pathogen richness (Table 3 and. Figure S4). Importantly, a strong rank correlation was also found for the full-length *SERPINB11* variant (E90-T181-P303 haplotype, p-value<0.0001). Overall, these data provide support for a slight overrepresentation of the full-length *SERPINB11* in geographic areas with a pastoral mode of subsistence, dry, polar and humid-tropical climates, and greater diversity of pathogens (Figure 6).

**Table 3:** SNP association with pathogen richness.

GENE	SNP	Kendall's rank correlation	
		τ (Kendall's coefficient)	*p-value*
***SERPINB11***	rs4940595	0.354	*0.0002*
	rs1395267	0.299	*0.0018*
	rs1395268	−0.207	*0.0306*
	rs1506418	0.342	*0.0004*
	Haplotype*	0.369	*0.0001*
***ABO*** •	rs2073824	−0.353	*0.0002*
***AQP3*** •	rs2228332	0.34	*0.0004*
***CD44*** •	rs2421826	−0.29	*0.0024*
	rs1547059	−0.288	*0.0026*
***CD55*** •	rs6700168	−0.373	*<0.0001*
***C1GALT1*** •	rs10487590	−0.413	*<0.0001*
***ERMAP*** •	rs11210729	0.33	*0.0005*
***FUT2*** •	rs485186	0.377	*<0.0001*
	rs602662	0.376	*<0.0001*
	rs504963	0.372	*0.0001*
***GCNT2*** •	rs9466910	0.284	*0.0029*
	rs9466912	0.284	*0.0029*
***GYPC*** •	rs7589096	0.366	*0.0001*
	rs4143022	0.314	*0.001*
	rs4663038	0.343	*0.0003*
	rs17741574	0.336	*0.0004*
	rs13034269	0.371	*0.0001*
	rs6568	0.371	*0.0009*
	rs7589096	0.366	*0.0001*
***SLC4A1*** •	rs2072081	0.395	*<0.0001*
	rs2074108	0.375	*<0.0001*
***SLC14A1*** •	rs900971	0.491	*<0.0001*
	rs692899	−0.296	*0.002*
	rs10853535	−0.402	*<0.0001*
	rs566309	−0.318	*0.0009*
	rs6507641	−0.387	*<0.0001*

*Haplotype E90-T181-P303.

•Blood Group Antigen genes significantly associated with pathogen richness [33]. ***ABO***: ABO blood group; ***AQP3***: Aquaporin 3; ***CD44***: CD44 antigen; ***CD55***: CD55 antigen; ***CIGALT1***: Core 1 synthase, glycoprotein-N-acetylgalactosamine 3-beta-galactosyltransferase, 1; ***ERMAP***: Erythroblast membrane-associated protein; ***FUT2***: Fucosyltransferase 2; ***GCNT2***: glucosaminyl (N-acetyl) transferase 2, I-branching enzyme; ***GYPC***: Glycophorin C; ***SLC4A1***: Solute carrier family 4, anion exchanger, member 1; ***SLC14A1***: Solute carrier family 14, member 1.

### Phylogenetic-based tests of selection

To investigate the long-term evolution of *SERPINB11*, we performed a series of phylogenetic analyses using the coding sequences of eight primate species: *Homo sapiens, Pan troglodytes*, *Gorilla gorilla*, *Pongo pygmeus*, *Macaca mulatta*, *Papio anubis*, *Colobus guereza*, and *Callithrix jacchus*; and the sequence of *Canis lupus familiaris*, which was used as an out-group. To capture most of the human variation, we performed independent analyses using four alternative sequences (Table 4). To explore the nature of the selective pressures acting on *SERPINB11*, we calculated d*_N_*/d*_S_* ratios (ω; *d_N_* and *d_S_* correspond to non-synonymous and synonymous substitution rates respectively) assuming opposite evolutionary scenarios. None of the likelihood rates tests (LRTs) performed with *branch* and *site* models (see Materials and Methods) yielded significant results. Overall, the low estimates obtained for the entire phylogeny (∼0.35) suggest a conserved evolution of *SERPINB11*. Conversely, in the human lineage, ω values range from 0.57 to 0.97 depending on the amino acid composition (Table 4). In order to test the possibility that some codon positions of *SERPINB11* are evolving under positive selection, we applied the *branch-site* model (see Materials and Methods) to the four independent phylogenies.Even though the vast majority of sites are constrained or neutrally evolving, a few amino acid positions (2–3%) were likely to be under positive selection in *SERPINB11* (Table 4). The SERPINB11 amino acids identified with higher probabilities of being positively selected in humans were the codons 90, 148, 181, 188, 303 (human polymorphic sites)194 and 253 (human fixed positions).

**Table 4:** Phylogenetic-based test of selection for *SERPINB11*.

	M0^a^	Free-ratio^b^	Branch site model – *foreground* ω (proportions)^c^					
Sequence (variable positions)	ω	ω	Site classes^d^					Positively selected sites
			0	1	2a	2b	p-value	(Posterior Probabilities)
**SERPINB11b**			0.095	1.000	47.773	47.773	<0.05	90; 181; 194; 253; 303
(A51-E90-T148-T181-R188-P303)	0.36	0.87	(68.2%)	(29.8%)	(1.4%)	(0.6%)		(0.93; 0.71; 0.74; 0.72; 0.70)
**SERPINB11c**			0.096	1.000	30.781	30.781	<0.05	90; 148; 181; 188; 194; 253; 303
(A51-E90-M148- T181-R188-P303)	0.36	0.97	(67.5%)	(29.2%)	(2.3%)	(1.0%)		(0.95;0.77 ;0.76; 0.76;0.79;0.79; 0.76)
**SERPINB11Xa**			0.090	1.000	1.000	1.000	NS	NA
(A51-X90-T148-A181-W188-P303)	0.35	0.63	(39.4%)	(17.6%)	(29.7%)	(13.3%)		
**SERPINB11Xb**			0.084	1.000	1.289	1.289	NS	NA
(E51-X90-T148- A181-W188-S303)	0.35	0.57	(54.9%)	(25.4%)	(13.5%)	(6.2%)		

a Model assuming a single ω value for all lineages in the phylogeny;

b Model assuming diferent ω values for each lineage in the phylogeny – value obtained for the human lineage;

c Model assuming a different ω value in *foreground* branch (in this case human lineage);

d Sites Classes: 0 – sites under constrains; 1 – neutral sites; 2a – constrained sites under positive selection in the *foreground* branch; 2b – neutral sites under positive selection in the *foreground* branch. NS – non-significant; NA – not applicable.

### Protein modeling

**Figure 7:**
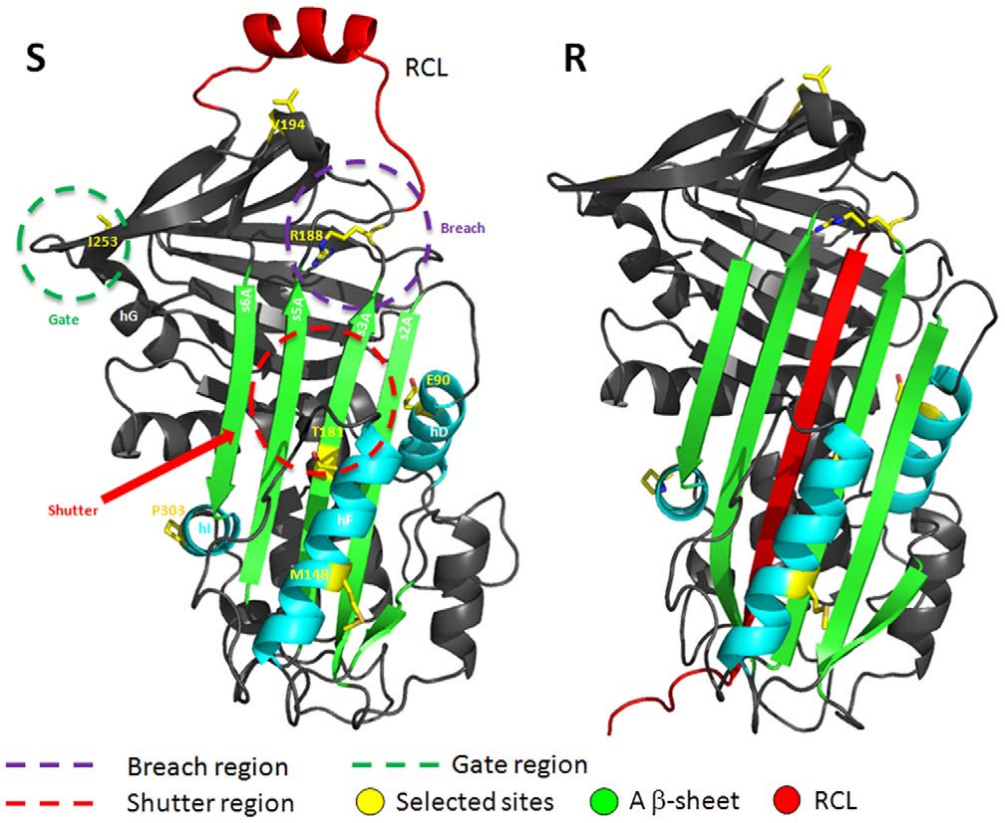
Comparative modeling structures of the SERPINB11c sequence. The two main conformational stages of the protein are presented – Stressed (S) and Relaxed (R). The image indicates the most important regions for inhibitory function – RCL, breach, shutter, and gate [3], [4]. Positive selection sites are highlighted and nearby elements of the secondary structure are indicated – α helix (hD and hF) and β strand (s2A, s3A, s5A, and s6A). This figure was generated using PyMOL [93] (PyMOL. DeLano Scientific, San Carlos).

To better understand the implications of the seven amino acid replaced (90, 148, 181, 188, 194, 253, and 303), we used comparative modeling methods to build three-dimensional structures of *Homo* (non-inhibitory) and *Pan* (probably inhibitory) SERPINB11 sequences (Figure 7).

In all the models generated, the amino acids at positions 90 (L*_Pan_* to E*_Homo_*), 148 (T*_Pan_* to M*_Homo_*) and 303 (S*_Pan_* to P*_Homo_*) are placed into helices D, F and I (hD, hF and hI) respectively. These helices are near the A β-sheet (strands s2A, s3A, s5A, and s6A – see Figure 7), which undergoes major structural changes during insertion of RCL in the shutter region. Therefore, substitution of other residues with different stereochemical properties can easily promote the destabilization of the helices where they are located. Moreover,as experimentally reported, L90P and S303P substitutions can account for major structural changes capable of affecting the dynamics of the RCL insertion in the A β-sheet [15], [39].

Two other interesting substitutions at positions 181 (A*_Pan_* to T*_Homo_*) and 188 (W*_Pan_* to R*_Homo_*) – are located respectively in the shutter and breach regions of SERPINs. Both amino acids' positions are packed in the protein medium, and consequently the replacement of nonpolar residues (A*_Pan_* and W*_Pan_*) by polar residues (T*_Homo_* and R*_Homo_*) may also affect the stability of shutter and breach regions of the protein.

Finally, two other substitutions that may contribute to the inability of human SERPINB11 to inhibit proteases are observed in positions 194 (V*_Pan_* to E*_Homo_*) and 253 (T*_Pan_* to I*_Homo_*), which are located in solvent-exposed regions. Placing polar residues in such highly hydrophobic regions of the protein may significantly affect its stability and, as mentioned before, influence its function.

Together, the substitutions described above appear to interfere directly or indirectly with the A β-sheet arrangement (breach, shutter, and gate regions) [3], [4]. These changes can contribute to a major destabilization of the protein folding that may ultimately influence the structural rearrangements necessary for the protein to undergo the S-to-R transition, thus affecting SERPINB11's ability of to inhibit proteases.

## Discussion

In recent years, the availability of large catalogues of human genetic variability has allowed perliminary insights into the extent of selection in the human genome. However, only a limited number of genes overlap across independent studies, which supports a cautious interpretation of results along with the need for in-depth follow-up studies of likely targets of selection [40]–[42]. Careful scrutiny of several genes is providing a new perspective on how natural selection may act on preexisting variants [43]–[45]. Our investigation of *SERPINB11* seems to support a complex and distinctive evolutionary history that cannot be explained by neutral scenarios or by the selective advantage of a newly arisen mutation.

Identification of *SERPINB11* as a potential candidate gene for selection in the YRI population occurred through a GWS based on the iHS statistic and HapMap phase II [16]. The iHS statistic has the capacity to identify the hallmarks of recent positive selection by comparing the extent of homozygosity in haplotypes that are defined by opposite SNP alleles [16], [17]. Although a selected site is not required to have a significant iHS score, the site should at least be surrounded by multiple SNPs with strong values (|iHS|>2) [16], [17]. Note that iHS signals found in the YRI are frequently more reliable, narrower, and older than those in other populations [16], [17], [41]. However, from the early beginnings of the reevaluation of HapMap haplotypes, *SERPINB11* patterns seemed difficult to reconcile with a standard selective scenario. Recognition of a single long-range haplotype was complicated by the presence of a strong recombination hotspot; and the identification of a likely selected site was problematic because the alleles with potential functional importance had non-significant values (−1.02<iHS<−0.35).

Understanding the biological significance of the phasing of alleles E90, T181, R188, and P303; and rs953696T, and rs953694C (miRs binding sites) with implications in gene translation and non-inhibitory activity provides an alternative interpretation to the *SERPINB11* selective signature. Given the possibility that these six derived alleles may define a common and long-range haplotype connecting the two clusters of SNPs with significant iHS scores, we proposed this full-length *SERPINB11* variant as the likeliest allele targeted by selection. Although these findings contrast with known examples of positive selection, in which single allele variants trigger the adaptive response, there are several reports in which configurations of tightly linked alleles do appear to have been driven by selection [20], [46].

When statistics of sequence variation are taken into account, the assumption of a standard selective sweep for *SERPINB11* becomes even less likely. The positive Tajima's D and the high diversity disclosed by *SERPINB11* in the YRI place this gene in the category of genes cited as examples of genes under long-term balancing selection [33], [47], [48]. However, long-term balancing selection can be mimicked by other adaptive scenarios, such as selection on standing variation, which may produce an excess of intermediate-frequency variants in an appreciable number of cases [49]. Such a model of directional selection is based on the assumption that adaptation may exploit the standing genetic pool of a population in such a way that shifts of selective pressures favor previously neutral or weakly deleterious variants [49]–[53].

In the early Holocene, major ecological changes (including the end of the glacial period and the onset of agriculture and animal husbandry) had a great impact on the pathogen burden in human populations. Simultaneously, the social modification from small groups of hunter-gatherers to more densely settled communities further contributed to a faster dispersal of infectious agents and thus to more severe outbreaks of disease. Those periods, probably entailed significant challenges to fitness, forcing rapid adaptive responses that could have been resolved immediately if beneficial variants already existed in the population [51]. For these reasons, we propose that the large environmental changes starting ∼12,000 years ago triggered the emergence of a *SERPINB11* variant that already existed in human populations, possibly by conferring a selective advantage related to host-pathogen interactions. Several lines of evidence support this hypothesis:

- first, the occurrence in YRI and other populations of a divergent haplotype, characterized by several tightly linked functional mutations unlikely to have originated simultaneously;
- second, the more recent T_MRCA_ of the full-length *SERPINB11* variant (16,500 years), compared to the age estimates of *SERPINB11* alleles (0.24 MY and 0.88 MY), as if the former had recently been driven to higher frequencies;
- third, the findings of positive correlations among *SERPINB11* allele frequencies (E90, T181, and P303), distributions, and pathogen richness with the same order of magnitude as other genes already known to determine innate resistance to several pathogens [33]; and
- fourth, the significant association between the pastoral subsistence variable and a trend towards higher frequencies of *SERPINB11* alleles (E90 and T181), as if they confer an advantage in environments with higher risk of infectious diseases transmission from domesticated animals to humans.

Assuming a selective advantage through a role in host-pathogen interactions might presuppose the same pathogen burden during the last 12,000 years but, overall, our findings for *SERPINB11* concur with the proposal of Hancock and colleagues [36] in which common variants, showing subtle differences across populations and correlated with environmental variables, are likely to play important roles in the architecture of human adaptation as a result of selection on standing variation.

More importantly, a significant association can be established between *SERPINB11* and a disease trait – as observed by Hancock and colleagues [36] for other SNPs strongly correlated with environmental variables. The Wellcome Trust Case Control Consortium study of patients with Crohn's disease detected a significant association with the X90E polymorphism (rs4940595; p-value 0.00063), as well as a 3% reduction in the frequency of the E90 variant [54]. Crohn's disease is a multifactorial inflammatory disease instigated and perpetuated by bacterial infections; it affects mainly ileum and cecum but may involve the entire gastrointestinal tract [55], [56]. Note that *SERPINB11* expression has been detected in the respiratory and upper gastrointestinal tract (lung, trachea, pharynx, esophagus, and mouth) and several other organs connected to innate immune functions (tonsil and placenta), which differs greatly from the ubiquitous expression of mouse *Serpinb11* [15]. In fact, the *SERPINB11* candidate variant comprises two sites predicted to determine the binding of at least three miRs. These molecules are currently known to participate actively in gene silencing of both normal and abnormal cells – making it attractive to speculate about the role of miRs in shifting from a ubiquitous to a more specialized pattern of expression in the arms race against pathogens.

Phylogenetic tests provide an independent line of evidence favoring the adaptive hypothesis for *SERPINB11*, disclosing a significant evolutionary impact of replacements at codon positions 90, 148, 181, 188, 194, 253, and 303 in humans. Consistent with comparative models of chimpanzee and human SERPINB11 structures, these replacements can exert a combined effect on the A β-sheet, which is likely to disturb the prototypical SERPIN folding, impairing the S-to-R transition and the capacity for permanent inhibition of proteases.

Conversely, primates show an overall conserved evolution of *SERPINB11*. While most nonhuman primates have a leucine residue encoded by a TTA codon at position 90 (*Papio*, *Colobus*, and *Macaca, Pongo*, *Gorilla*, and *Pan*), humans carry either a stop codon, TAA (×90), or a glutamic codon, GAA (E90). Collectively, these data indicate a more parsimonious hypothesis: the initial pseudogenization of *SERPINB11* followed by a gene *resurrection* event (TTA→TAA→GAA). To our knowledge, *SERPINB11* and the immunity-related GTPase M (*IRGM*) [57] represent the only examples to date of *resurrected* genes in recent primate evolution – in humans (<5–6 MY) and in great apes (<20 MY) respectively. In the *Homo* lineage, the ancient pseudogenization of *SERPINB11* is confirmed by the recently released Neanderthal and Denisova sequences [58], [59], all bearing the ×90 alelle (as well as other pseudogene associated alleles). These give a minimum time frame of 600,000–800,000 years for the origin of the pseudogene, which corresponds to the time at which Neanderthal and Denisova diverged from modern humans [59]–[61],

Given that genes within gene families have some degree of redundancy, an ancestral loss of *SERPINB11* might have had little impact on fitness due to possible buffering by paralogous genes. If such a phenomenon could underlie the fixation of a weak deleterious variant in a species of reduced effective size, loss of *SERPINB11* might also have a selective advantage. The “less is more” hypothesis posits that nonfunctional mutations are an important source of evolutionary adaptations [62], a view supported by other examples of polymorphic pseudogenes [63]–[65]. However, it may be virtually impossible to discriminate between the two alternative hypotheses for an ancient event of pseudogenization occurring 5.4 to 0.6 MY ago.

Despite strong evidences for *SERPINB11*'s role in host-pathogen interactions these are based strictly on genetic data and require further molecular and cellular analysis to determine the underlying mechanisms. Importantly another SERPIN was recently shown to exert broad antimicrobial activity through the permeabilization of bacteria cell walls [66] and small peptides resulting from the proteolysis of SERPINs were shown to have similar proprieties [67], [68].

In summary: We have uncovered a complex selective signature of *SERPINB11*, which may well represent one of the most extraordinary examples of molecular evolution in humans. This gene, likely to have been conserved throughout evolution, was lost in humans. However. the accumulation of a series of new mutations contributed to the eventual appearance of a modified gene which – under a new set of environmental conditions, has emerged through selection as a beneficial allele.

## Materials and Methods

### DNA samples

Sequence variation was surveyed in a subset of 20 YRI belonging to the sample collection of the International HapMap Project Phase I/II (NA18501, NA18853, NA18870, NA18913, NA19092, NA19141, NA19144, NA19152, NA19203, NA19207, NA19210, NA19209, NA18522, NA18855, NA18856, NA19140, NA19160, NA19201, NA19200, NA19223).

To perform phylogenetic evolutionary analysis of *SERPINB11*, we retrieved from Genbank (http://www.ncbi.nlm.nih.gov/) the following coding sequences with the accession numbers: XM_523958 for *Pan troglodytes*; XM_001091618.1 for *Macaca mulatta*; DP000514 for *Papio anubis*; DP000562 for *Callithrix jacchus*; and XM_541073 for *Canis lupus familiaris*. The sequence of *Colobus guereza* was reconstructed from raw data downloaded from BLAST Trace Archives (http://blast.ncbi.nlm.nih.gov/Blast.cgi), and *Pongo pigmeus* was identified using the BLAT tool (http://genome.ucsc.edu/cgi-bin/hgBlat). To obtain coding data for *Gorilla gorilla*, we sequenced the EB(JC) sample purchased from the European Collection of Cell Cultures (ECACC). For *Homo sapiens*, we used four distinct sequences to capture most common variations and haplotypes. Two sequences corresponding to the active *SERPINB11* were used: AF419954.1 (*SERPINB11b*), and AF419955.1 (*SERPINB11c*). Two sequences corresponding to the *SERPINB11* pseudogene were also used. The first sequence differs from the reference sequence (NC_000018.8) at codon 373 (rs953695T→G) and was named *SERPINB11Xa*. The second sequence differs from the reference sequence at codons 51 (rs1395268C→A), 293 (rs1395266T→C), and 303 (rs1395267T→C), and was designated *SERPINB11Xb*.

### Polymerase Chain Reaction and Sequencing

Primers were designed on the basis of reference assembly for chromosome 18 (NC_000018.8) between bases 59519057 and 59541613 (http://www.ncbi.nlm.nih.gov/). All nucleotide positions in this article are numbered according to this sequence. Sequencing was performed using the ABI BigDye Terminator version 3 cycle sequencing chemistry (Applied Biosystems, Foster City, CA), and electrophoretic analysis was done on an ABI 3130 automated sequencer. All human sequences were assembled and analyzed using the Phred-Phrap-Consed package [69]. All putative polymorphisms and software-derived genotype calls were visually inspected and confirmed using Consed. Details about PCR and sequencing conditions are available from the authors upon request.

### Statistical Analysis

Phased haplotypes from the International HapMap Project Phase II (release 21) for a 200 kb region centered on *SERPINB11* in the YRI population were downloaded from the HapMap Web site (http://hapmap.ncbi.nlm.nih.gov/). Haplotype data were then annotated with additional SNP information regarding ancestral allele state and potential selected sites. Ancestral allele state was retrieved from dbSNP (http://www.ncbi.nlm.nih.gov/) and potential selected sites were identified using the Haplotter application (http://hg-wen.uchicago.edu/selection/haplotter.htm). A |iHS|>2 threshold, corresponding to the top 5% of iHS values for the entire genome [16], was used to identify the potential selected sites.

LD analysis was applied to HapMap data using Haploview software [70] and haplotype blocks were identified through the implemented method of Gabriel and colleagues [71].

Statistics of polymorphism data were calculated using the applications SLIDER (http://genapps.uchicago.edu/slider/index.html) and MAXDIP (http://genapps.uchicago.edu/labweb/index.html). Haplotypes of *SERPINB11* were inferred by using the program PHASE 2.02 [72], [73], where SNPs previously inferred by HapMap Phase II were entered as known-phase.

To assess the statistical significance of Tajima's D, we ran 100,000 coalescent simulations [74] using estimates of the ρ and θ_W_ parameters calculated for our data using MAXDIP and SLIDER. Simulations were produced assuming distinct demographic models described elsewhere [26], [27], [64], [75]. For each model, we obtained null distributions of summary statistics and calculated their 97.5^th^ percentiles.

The neutral parameter of the maximum likelihood of θ (θ_ML_) and T_MRCA_ were estimated by a coalescent method implemented in GENETREE version 9 [28]. Once GENETREE assumed no recombination, we had to subdivide *SERPINB11* data into two regions, upstream and downstream of the recombination hotspot. In addition, we had to exclude from the analysis a few incompatible sites and haplotypes. Time, scaled in 2Ne generations, was derived from θ_ML_ = 4Neμ. The estimate of the mutation rate per gene per generation (μ) was calculated from the number of nucleotide substitutions per site (Dxy) averaged between human and chimpanzee reference sequences, calculated in DnaSP v.4.9 [76]. Time estimates in generations were converted into years using a 25-year generation time. Human/chimpanzee divergence was assumed to have occurred about 5.4 million years ago [77].

To estimate the T_MRCA_ of the full-length *SERPINB11* variant, we used the measure of the DHS, implemented in the software for fine-scale mapping, DHSMAP (version 2.0) [78].

The 16 haplotypes carrying the candidate variant (cases) were separated from remaining haplotypes (controls) and a maximum likelihood approach was used to calculate a LD statistic (τ) later translated into the time in generations to the ancestor of the full-length *SERPINB11* variant (1/τ). The genetic distances per marker were inferred from the chromosome 18 HapMap phase II recombination rates (http://hapmap.ncbi.nlm.nih.gov/) and a mutation rate of 2.5×10^−8^ per marker was assumed [79].

### Correlation with pathogen richness

Genotype data from 650,000 tag SNPs for the HGDP panel [35] were downloaded from the Web site: http://hagsc.org/hgdp/files.html. To determine the pathogen richness, we considered the list of intracellular pathogens (viruses, bacteria, and protozoa) and the indications of Prugnolle and colleagues [38]. The matrix of pathogen presence/absence in the 21 countries from HGDP populations was extracted from the GIDEON database (http://gideononline.com). The correlations between pathogen richness and SNPs allele frequency were determined by Kendall's rank correlations implemented in the StatView statistical package, version 5.0.

### Phylogenetic analysis of selection

Four alternative phylogenies, differing in the human sequences used, were built using the maximum likelihood method implemented in the DNAml program from the Phylogeny Inference Package (PHYLIP – http://evolution.genetics.washington.edu/phylip.html). Except for a shift in *Pan* and *Gorilla* sequences, there was a close agreement between gene tree and species tree [80], [81]. Maximum likelihood estimates of *d_N_/d_S_* (ω), were carried out using the *codeml* program from the software package Phylogenetic Analysis by Maximum Likelihood - PAML version 4.2 [82], [83]. The following LRTs were performed: 1) the *branch* model [84], [85], which compares a single ω value obtained for all lineages (M0) with a model assuming different ω values for each lineage (free-ratio); 2) the *site* models [86], [87], which allow the ω value to vary among sites of the protein and compare models of neutrality with positive selection (M1-M2 and M7–M8); and 3) the *branch-site* model, which assumes that branches in the phylogeny are divided *a priori* into *foreground* and *background* and allows ω to vary both among sites in the protein and across branches [88]–[90]. Values of ω>1 were considered as evidences of positive selection and the values of ω<1 were regarded as an indicative of purifying selection. The significance of each nested model was obtained from twice the variation of likelihoods (2Δl) using a χ^2^ statistic. For the *branch-site* model, comparisons with critical χ^2^ were carried out as described [90]. The Bayes empirical Bayes (BEB) [89] was used to calculate posterior probabilities of site classes, in order to identify sites under positive selection for the significant LRTs.

### Comparative Modeling

The generated models of S and R structures of SERPINB11 for *Homo* (SERPINB11c) and *Pan* (XM_001091618) were based on the three-dimensional structures of paralogous proteins. In the modeling, the structures of chicken ovalbumin protein (pdb reference: 1OVA, 1UHG and 1JTI)) and SERPINB3 (pdb reference: 2ZV6) were used Each protein6 showed a sequence identity of approximately 37% with SERPINB11.

Modeller 9v6 [91] was used in all modeling tasks. Sequence and structural alignments were carried out using ALIGN2D and ALIGN3D features of Modeller and optimized through several cycles of comparative modeling. In the last cycle, 200 different models were generated and the one with the lowest value for the Modeller's objective function was chosen as the most representative of each protein. The optimization procedure used in generating the four different structural models was guided by a stereochemical analysis of the models performed by the program PROCHECK [92].

## Supporting Information

Figure S1 **LD plot of HapMap phase II YRI data centered on the** ***SERPINB11*** **region.** The image was constructed using *Haploview* 4.1 software. The triangular units designate LD blocks. The degree of LD between pairs of markers is indicated by the |D′| statistic (|D′| = 1, bright red; |D′|>1, shades of red).
(TIF)

Figure S2 **Empirical distribution of Tajima's D and π built using the 316 genes surveyed by SeattleSNPs (** http://pga.gs.washington.edu/ **).** Genes within the upper extreme (97^th^) of the distribution are marked in grey. Gene classes with values close to survey genes are indicated by *SERPINB11* gene name. **A**–**C**: YRI sample – SeattleSNPs panel 2; **D–F**: All African descend samples – SeattleSNPs panels 1 to 3. *ABO*: ABO blood group; *CD151*: CD151 antigen; *CYP4F3*: Cytochrome P450, family 4, subfamily F, polypeptide 3; *FLG1*: Fibrinogen-like 1; *FUT2*: Fucosyltransferase 2; *GPR109G*: G protein-coupled receptor 109B.
(PDF)

Figure S3 **A** - Linkage Disequilibrium (LD) plot of HapMap phase II for YRI, CEU and CHB+JPT data centered on *SERPINB11* region of chromosome 18. The image was built using *Haploview* 4.1 software. The triangular units designate LD blocks. The degree of LD between pairs of markers is indicated by the |D′| statistic (|D′| = 1 bright red; |D′|>1 shades of red) LD blocks overlapping between populations are indicted. **B** – Haplotype structure of LD blocks reconstructed by median-joining networks (http://www.fluxus-engineering.com/sharenet.htm).
(PDF)

Figure S4 **Correlation between pathogen richness and derived allele frequency for** ***SERPINB11*** **SNPs (rs1395268, rs4940595, rs1506418 and rs1395267).** Worldwide frequency variation (images retrieved from HGDP selection browser - http://hgdp.uchicago.edu/cgi-bin/gbrowse/HGDP/).
(PDF)

File S1 **Annotated haplotypes from HapMap phaseII - YRI sample.** Haplotypes defined by cluster of SNPs with significant iHS scores are limited in red. Single long-range haplotype linking the two blocks is limited in black.
(XLSX)

File S2 **Annotated haplotypes from HapMap phaseII - YRI sample, CEU sample and JPT+CHB sample.**
(XLSX)
