# Supplementary figures and images for: Loss and Gain of Function in SERPINB11: An Example of a Gene under Selection on Standing Variation, with Implications for Host-Pathogen Interactions
Source: PLoS One. 2012 Feb 29;7(2):e32518. doi: 10.1371/journal.pone.0032518 (PMC3290568; doi:10.1371/journal.pone.0032518)

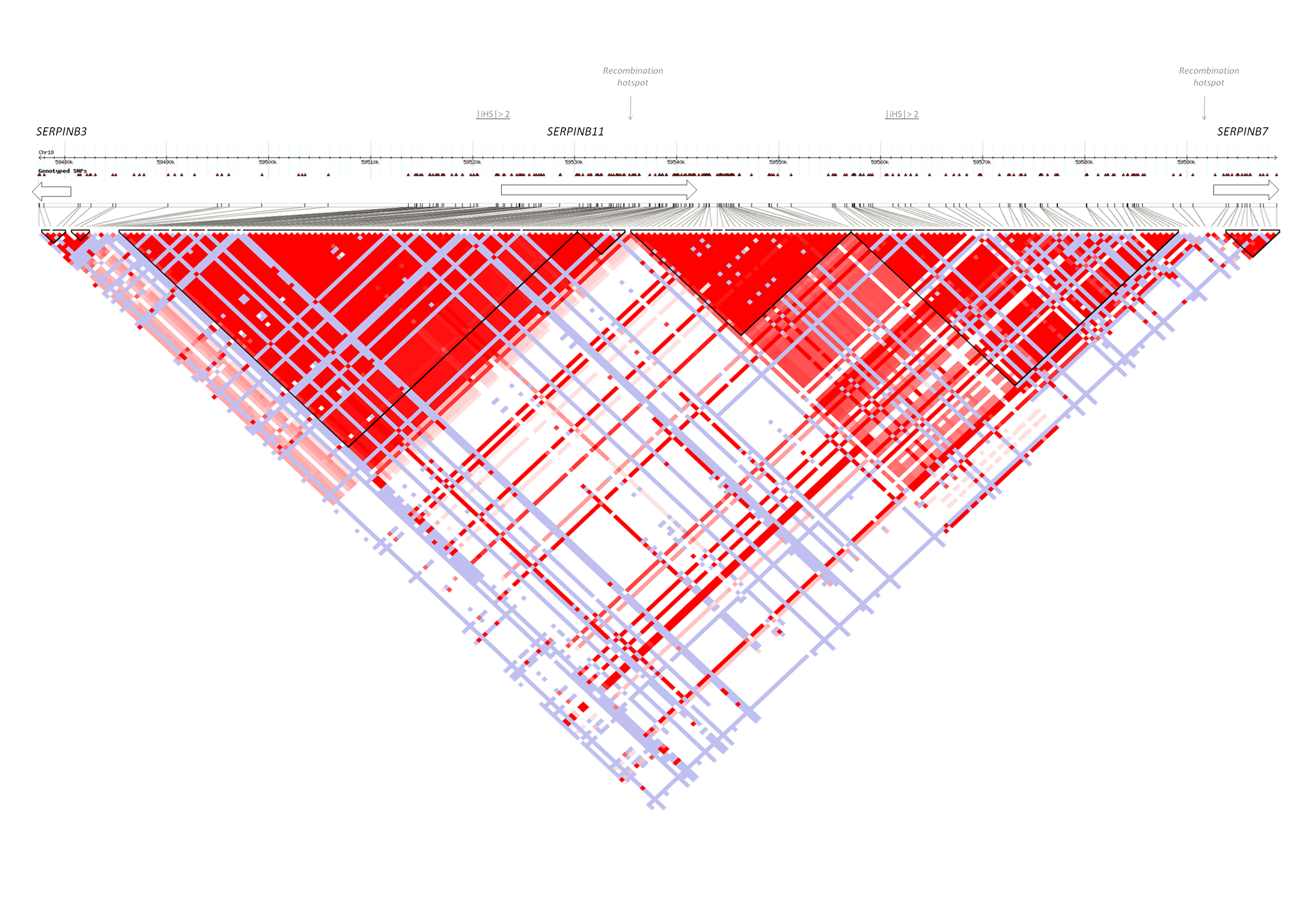

Supplement: Figure S1 — LD plot of HapMap phase II YRI data centered on the SERPINB11 region. The image was constructed using Haploview 4.1 software. The triangular units designate LD blocks. The degree of LD between pairs of markers is indicated by the |D′| statistic (|D′| = 1, bright red; |D′|>1, shades of red). (TIF) [file pone.0032518.s001.tif]
