# Supplementary material for: Loss and Gain of Function in SERPINB11: An Example of a Gene under Selection on Standing Variation, with Implications for Host-Pathogen Interactions
Source: PLoS One. 2012 Feb 29;7(2):e32518. doi: 10.1371/journal.pone.0032518 (PMC3290568; doi:10.1371/journal.pone.0032518)
Supplement: Figure S2 — Empirical distribution of Tajima's D and π built using the 316 genes surveyed by SeattleSNPs ( http://pga.gs.washington.edu/ ). Genes within the upper extreme (97th) of the distribution are marked in grey. Gene classes with values close to survey genes are indicated by SERPINB11 gene name. A–C: YRI sample – SeattleSNPs panel 2; D–F: All African descend samples – SeattleSNPs panels 1 to 3. ABO: ABO blood group; CD151: CD151 antigen; CYP4F3: Cytochrome P450, family 4, subfamily F, polypeptide 3; FLG1: Fibrinogen-like 1; FUT2: Fucosyltransferase 2; GPR109G: G protein-coupled receptor 109B. (PDF) [file pone.0032518.s002.pdf]

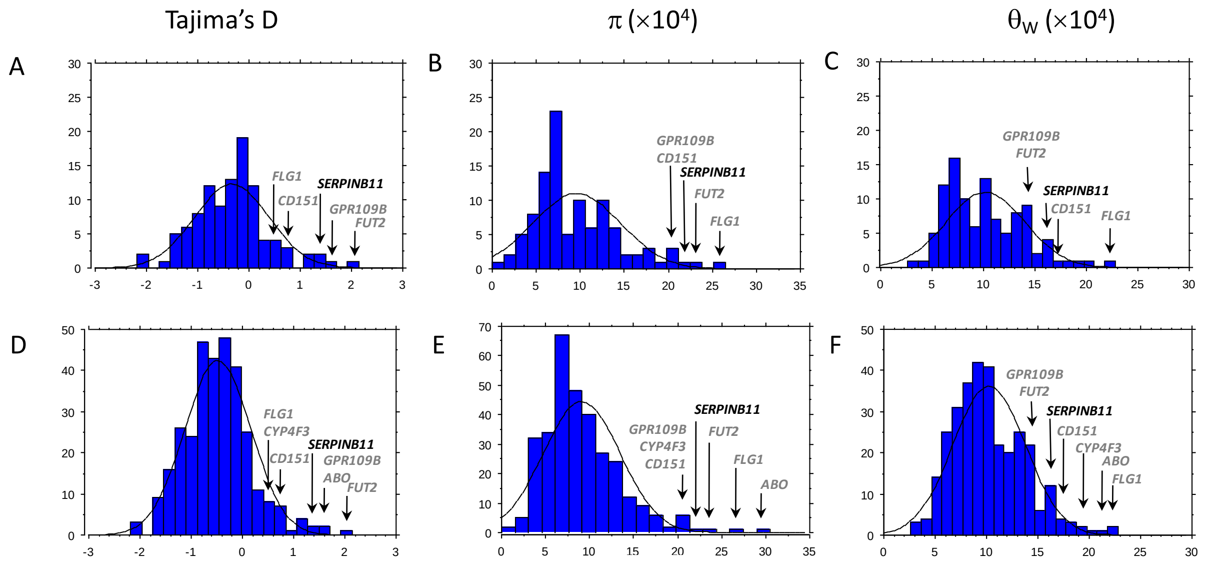

Figure S2: Empirical distributions of Tajima's D and π built using the 316 genes surveyed by SeattleSNPs (http://pga.gs.washington.edu/). Genes within the upper extreme (97th) of the distribution are marked in grey. Gene classes with values close to survey genes are indicated by *SERPINB11* gene name. **A - C**: YRI sample – SeattleSNPs panel 2; **D - F**: All African descend samples – SeattleSNPs panels 1 to 3. *ABO*: ABO blood group; *CD151*: CD151 antigen; *CYP4F3*: Cytochrome P450, family 4, subfamily F, polypeptide 3; *FLG1*: Fibrinogen-like 1; *FUT2*: Fucosyltransferase 2; *GPR109G*: G protein-coupled receptor 109B.
