# Supplementary material for: Loss and Gain of Function in SERPINB11: An Example of a Gene under Selection on Standing Variation, with Implications for Host-Pathogen Interactions
Source: PLoS One. 2012 Feb 29;7(2):e32518. doi: 10.1371/journal.pone.0032518 (PMC3290568; doi:10.1371/journal.pone.0032518)
Supplement: Figure S3 — A - Linkage Disequilibrium (LD) plot of HapMap phase II for YRI, CEU and CHB+JPT data centered on SERPINB11 region of chromosome 18. The image was built using Haploview 4.1 software. The triangular units designate LD blocks. The degree of LD between pairs of markers is indicated by the |D′| statistic (|D′| = 1 bright red; |D′|>1 shades of red) LD blocks overlapping between populations are indicted. B – Haplotype structure of LD blocks reconstructed by median-joining networks (http://www.fluxus-engineering.com/sharenet.htm). (PDF) [file pone.0032518.s003.pdf]

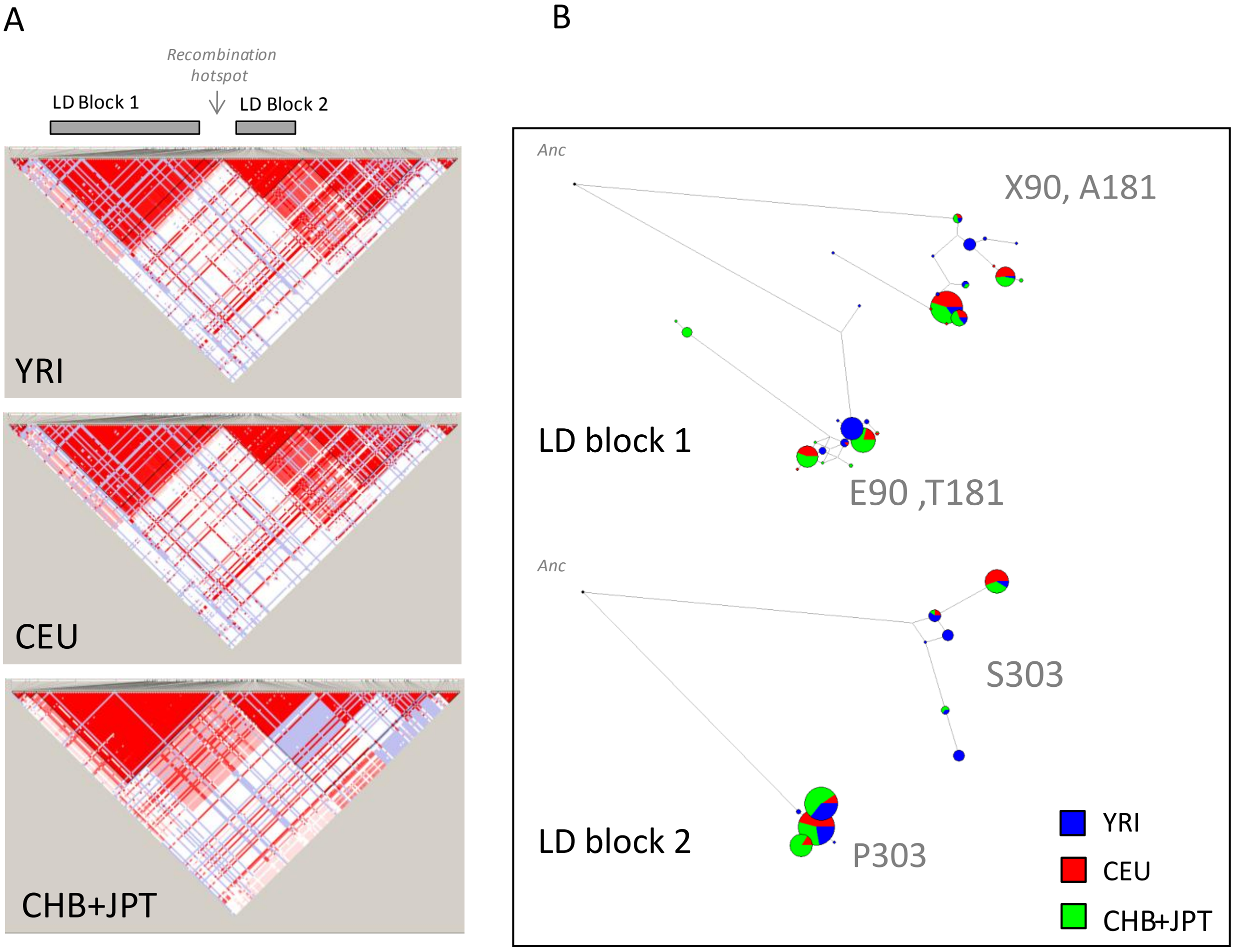


**Figure S3:** **A** - Linkage Disequilibrium (LD) plot of HapMap phase II for YRI, CEU and CHB+JPT data centered on *SERPINB11* region of chromosome 18. The image was built using *Haploview* 4.1 software. The triangular units designate LD blocks. The degree of LD between pairs of markers is indicated by the |D'| statistic (|D'| =1 bright red; |D'| >1 shades of red) LD blocks overlapping between populations are indicted. **B** – Haplotype structure of LD blocks reconstructed by median-joining networks (http://www.fluxus-engineering.com/sharenet.htm).
