# Supplementary material for: Loss and Gain of Function in SERPINB11: An Example of a Gene under Selection on Standing Variation, with Implications for Host-Pathogen Interactions
Source: PLoS One. 2012 Feb 29;7(2):e32518. doi: 10.1371/journal.pone.0032518 (PMC3290568; doi:10.1371/journal.pone.0032518)
Supplement: Figure S4 — Correlation between pathogen richness and derived allele frequency for SERPINB11 SNPs (rs1395268, rs4940595, rs1506418 and rs1395267). Worldwide frequency variation (images retrieved from HGDP selection browser - http://hgdp.uchicago.edu/cgi-bin/gbrowse/HGDP/). (PDF) [file pone.0032518.s004.pdf]

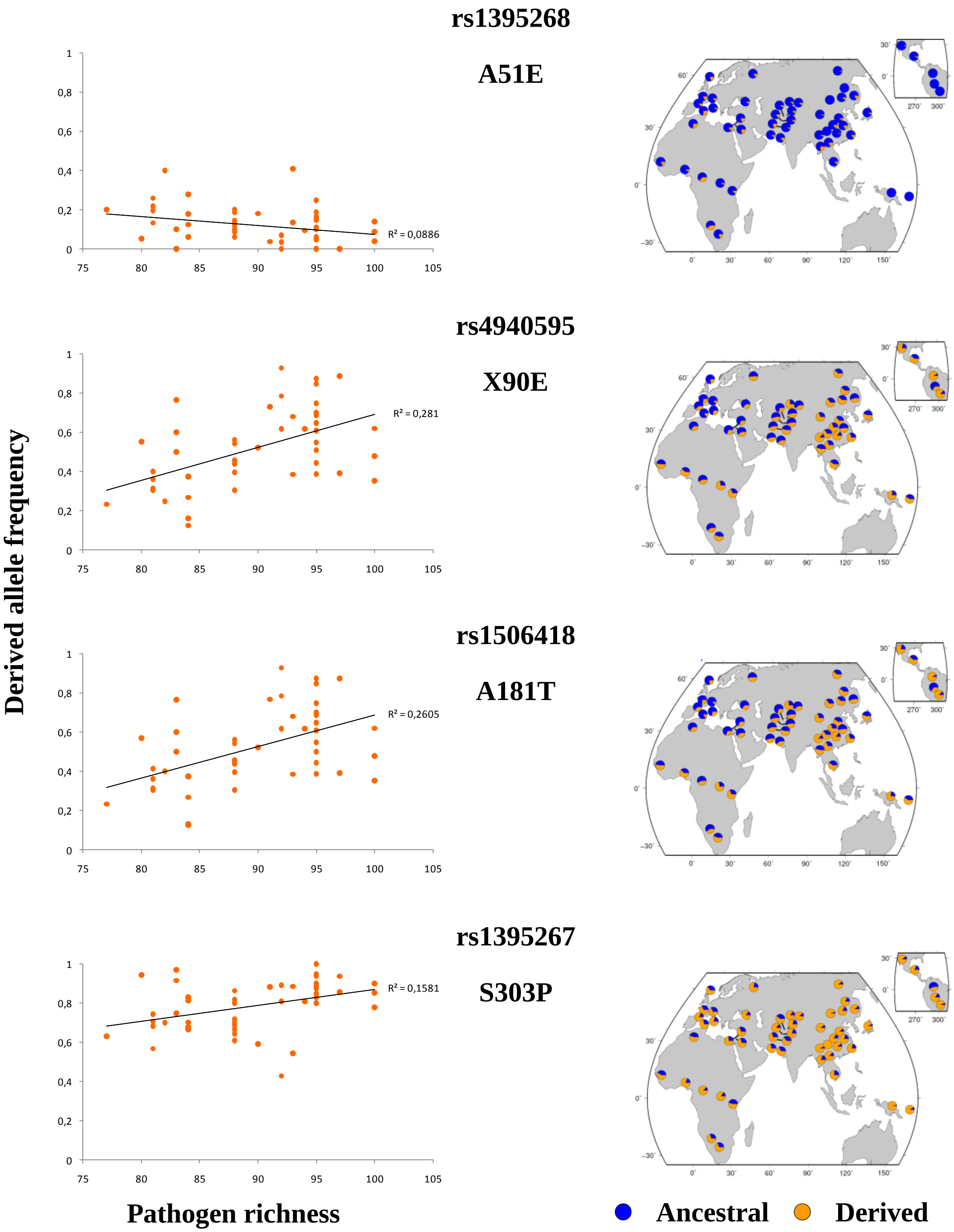

Figure S4: Correlation between pathogen richness and derived allele frequency for *SERPINB11* SNPs (rs1395268, rs4940595, rs1506418 and rs1395267). Worldwide frequency variation (images retrieved from HGDP selection browser - http://hgdp.uchicago.edu/cgi-bin/gbrowse/HGDP/)
